# Notes and News

**Published:** 1899-09-30

**Authors:** 


### NOTES AND NEWS.

(Collected and Communicated.)

The Harveian Oration will be delivered before the Royal College of Physicians on Wednesday, October 18th.

The Princess of Wales recently visited and was much interested in Dr. Finsen's Electric Light Institute for the Treatment of Lupus.

A league against tuberculosis is being organised in Spain. It originated in Valencia, where Dr. Moliner, professor of pathology at the University, has actively headed the movement.

A new quarterly is about to be issued entitled *Climate*. The subject is a wide one, and as it is edited by Dr. Harford-Battersby, who is in touch with missionaries all over the world, we may expect an interesting publication.

The value of inoculation for enteric fever is likely to be largely tested among the troops in India. Fifty-nine non-commissioned officers and men of the 2nd Battalion Gloucestershire Regiment, which left this month for India, voluntarily underwent inoculation before starting.

A Parliamentary paper has been issued this week showing in respect of Poor Law unions in England and Wales the number of certificates of successful primary vaccination received during the periods between January 1st and June 30th in the years 1898 and 1899. From this document it appears that in no fewer than 40 union-counties in England the figures for the 1899 period mentioned showed an increase over those for the 1898 period, and in some instances the growth was very striking. In only three union-counties were the 1899 figures lower than those of 1898, namely, in Devon, Durham, and York (North Riding). In seven of the Welsh union-counties the 1899 figures were in excess of those in 1898, but nowhere was the increase so striking as in England.

Dr. Clifford Allbutt, Regius Professor of Physic, Cambridge, will deliver the prizes to the students of St. Thomas's Hospital Medical School on Tuesday, October 3rd, at three p.m.

The report of the German Commission on Malaria, which appears in the *Deutsche Medicinal Wochenschrift*, will be read with much interest at the present time. The investigations are mainly devoted to the study of the kind of mosquito engaged in the dissemination of the disease.

The open-air treatment is being followed at the Sheffield Infirmary with great benefit to the patients. If it is found beneficial both in Sheffield and London to adopt this treatment, no other towns need fear to make the experiment also, as a preliminary step to establishing sanitoria in all parts of the country, in situations most favourable to the arrest of the disease.

At the meeting of the French Association for the Advancement of Science at Boulogne Professor Brouardel, the president, devoted a great portion of his opening speech to considering advances made in recent years in hygiene and sanitation, especially dwelling on the influence of Pasteur in this direction. He dwelt on the importance of the best endeavours being made to arrest the progress of tuberculosis.

An examination of the weekly returns issued by authority of the Registrar-General shows that with the continuance of cooler weather the deaths from diarrhœa in London, which had been 368, 322, and 233 in the preceding three weeks, further fell last week to 162, but they were still 54 above the corrected average. On the other hand, the deaths from diseases of the respiratory organs rose slightly, but were still below the corrected average. In scarlet fever, diphtheria, and enteric fever the usual autumnal increase in prevalence is showing itself.

An interesting letter from Dr. Donald Ross, who is investigating the relations between the malaria parasite and the mosquito at Sierra Leone, is published in the *Times*. He says: "We have now practically finished our work here. We have found: (*a*) That local species of Anopheles (mosquitoes) carry malaria; (*b*) That these species breed in a few stagnant puddles. These observations support my inaugural lecture at every point. . . . The practical results to be derived from the facts which we have obtained will depend solely on the Government and medical profession here. . . . . Of course, we could kill most of the Anopheles grubs here in a few hours with kerosene oil, and the thing will be done shortly after Ould arrives. But this won't be enough. It is necessary that the operations be continued systematically, and that some of the more dangerous puddles be drained. . . . . For many scientific reasons we have come to the conclusion that the truly malarial fever is caused here solely by the mosquito—probably entirely by the Anopheles species. We estimate, then, that most of the malarial fever here can be got rid of at almost no cost except of a little energy on the part of the local authorities."

